# A Special Section Including Selected Reviews From the ESF ‘Integrated Approaches for Functional Genomics’ Programme Workshop ‘Proteomics: Focus on Protein Interactions’

**DOI:** 10.1002/cfg.103

**Published:** 2001-10


					Comparative and Functional Genomics
A Special Section including selected reviews from the ESF `Integrated
Approaches for Functional Genomics' programme workshop
`Proteomics: Focus on Protein Interactions'
Edited by Gianni Cesareni
WORKSHOP ORGANISERS
Gianni Cesareni - University of Rome, Tor Vergata
Manuela Helmer Citterich - University of Rome, Tor Vergata
Benedetta Mattei - University of Rome, La Sapienza
The European Science Foundation (ESF) acts as a catalyst for the development of
science by bringing together leading scientists and funding agencies to debate, plan
and implement pan-European scientific and science policy initiatives.
ESF is the European association of 67 major national funding agencies devoted to
scientific research in 24 countries. It represents all scientific disciplines: physical and
engineering sciences, life and environmental sciences, medical sciences, humanities and
social sciences. The Foundation assists its Member Organisations in two main ways: by
bringing scientists together in its scientific programmes, EUROCORES, networks,
exploratory workshops and European research conferences, to work on topics of
common concern; and through the joint study of issues of strategic importance in
European science policy.
It maintains close relations with other scientific institutions within and outside Europe.
By its activities, the ESF adds value by cooperation and coordination across national
frontiers and endeavours, offers expert scientific advice on strategic issues, and provides
the European forum for science.

				

